# Evidence of Age Estimation Procedures in Forensic Dentistry: Results from an Umbrella Review

**DOI:** 10.3390/medicina60010042

**Published:** 2023-12-25

**Authors:** João Albernaz Neves, Luísa Bandeira Lopes, Vanessa Machado, João Botelho, Ana Sintra Delgado, José João Mendes

**Affiliations:** 1 Clinical Research Unit (CRU), Centro de Investigação Interdisciplinar Egas Moniz (CiiEM), Egas Moniz, CRL, 2829-511 Monte de Caparica, Portugal; 2 Orthodontics Department, Egas Moniz Dental Clinic (EMDC), Egas Moniz, CRL, 2829-511 Monte de Caparica, Portugal

**Keywords:** forensic dentistry, age estimation, dental maturation, umbrella review

## Abstract

*Background and objective*: Age estimation is an important tool when dealing with human remains or undocumented minors. Although the skull, the skeleton or the hand-wrist are used in age estimation as maturity indicators, they often present a lack of good conditions for a correct identification or estimation. Few systematic reviews (SRs) have been recently published; therefore, this umbrella review critically assesses their level of evidence and provides a general, comprehensive view. *Materials and methods*: Considering the review question “What is the current evidence on age determination approaches in Forensic Dentistry?” an electronic database search was conducted in four databases (PubMed, Cochrane, WoS, LILACS) up to December 2022, focusing on SRs of age estimation through forensic dentistry procedures. The methodological quality was analyzed using the measurement tool to assess SRs criteria (AMSTAR2). *Results*: Eighteen SRs were included: five of critically low quality, six of low quality, three of moderate quality and four of high quality. The SRs posited that Willems’ method is more accurate and less prone to overestimation; most methods seem to be geographically sensitive; and 3D-imaging and artificial intelligence tools demonstrate high potential. *Conclusions*: The quality of evidence on age estimation using dental approaches was rated as low to moderate. Well-designed clinical trials and high-standard systematic reviews are essential to corroborate the accuracy of the different procedures for age estimation in forensic dentistry.

## 1. Introduction

Age estimation is a key forensic and archeological element. Often useful for forensic identification of human remains, legal assistance involving minors or clinical diagnosis and planning [1,2,3,4], it is also helpful in mass migration and lack of valid identification [1,2,5]. Several methods have been developed to this end, among them, skeletal and dental development, sexual maturation or height/weight ratios [6,7].

Although the skull, the skeleton or the hand-wrist are used in age estimation as maturity indicators, they often present a lack of good conditions for a correct identification or estimation [8,9]. Teeth are the hardest human organs and are often found in adequate conditions [10,11,12]. Furthermore, dental measurements and indices are considered more useful and reliable, due to less variability during development as well as the greater resistance of teeth to systemic, environmental or destructive factors [6,13].

Estimating dental age may be achieved through several strategies depending on whether tooth development (around 20 years of age) or body development have been completed. On the one hand, methodologies based on teeth development are more accurate and with a smaller margin of error [8,14]. On the other hand, the biological age of the individual is being estimated, always understood in a period of time, with some level of precision, and according to the method used. Chronological age will be included, at best, in this age range [12,14].

Several systematic reviews have been published with numerous dental methods based on radiographic (panoramic radiographs or otherwise) and non-radiographic approaches, most of them only evaluate one or two methodologies. Considering the variety and the discrepancy of the methods, it is helpful to compare and summarize the evidence previously published regarding age determination in Forensic Dentistry. The purpose of this comprehensive review was to assess the existing evidence on age determination procedures in forensic dentistry. Our focus was twofold: to determine the quality of the evidence and the overall clinical accuracy of each procedure.

## 2. Materials and Methods

We followed the Preferred Reporting Items for Systematic Reviews and Meta-Analyses (PRISMA) guideline [15] (Supplementary File S1) and the guide for systematic reviews of systematic review [16]. The review protocol was approved a priori by all authors and registered on Open Science Framework (DOI 10.17605/OSF.IO/CPBZY).

The review question was: “What is the current evidence on age determination approaches in Forensic Dentistry?”.

Eligibility criteria

To answer the proposed research question, the inclusion criteria were: (1) systematic review (with or without meta-analysis); (2) addressing age determination in Forensic Dentistry; (3) absence of data duplication within the included studies in the meta-analysis. No restrictions on the year of publication or language were applied.

Information sources search

Four electronic databases were searched for electronic data: PubMed, Cochrane Database of Systematic Reviews, LILACS (Latin American scientific literature in health sciences) and Web of Science. The key words and subject headings were merged in accordance with the thesaurus of each of the databases and the subject headings were exploded, with the following syntax “((age determination) OR (age determination forensic) OR (age estimation) OR (dental age estimation) OR (forensic age estimation) OR (age estimation methods) OR (age prediction) OR (dental age prediction)) AND ((tooth) OR (teeth) OR (dental) OR forensic OR (forensic dentistry) OR (forensic odontology)) AND ((Systematic Review) OR (Meta-analysis))”. Grey literature searches were conducted in three appropriate databases (opensigle.inist.fr, https://www.ntis.gov/, https://www.apa.org/pubs/databases/psycextra, accessed on 22 November 2022).

Study selection

Two researchers (JAN and LBL) independently reviewed titles and abstracts. Agreement between the reviewers was assessed using kappa statistics. Any paper that was deemed to be potentially eligible by one of the two reviewers was ordered as a full-text article and screened independently by the reviewers. Disagreements were discussed with a third reviewer (JB).

Data extraction process and data items

Two reviewers (JAN and LBL) separately extracted the following: authors and year of publication, objective/focal question, databases scanned, number of studies included, type of studies included, main results and main conclusions. Any differences of opinion were resolved by discussion with a third reviewer (JB).

Risk of bias assessment

To determine the methodological quality of the included systematic reviews, two researchers (JAN and LBL) used the A Measurement Tool to Assess Systematic Reviews (AMSTAR 2) [16]. AMSTAR 2 is a comprehensive 16-item tool that ranks the overall methodological quality of a systematic review. Accordingly, the quality is ranked as follows: High means ‘Zero or one non-critical weakness’; Moderate means ‘More than one non-critical weakness’; Low means ‘One critical flaw with or without non-critical weaknesses’; and Critically low means ‘More than one critical flaw with or without non-critical weaknesses. The AMSTAR 2 online tool was used to calculate the AMSTAR quality score for each study. (https://amstar.ca/Amstar_Checklist.php, accessed on 11 February 2023).

## 3. Results

Study selection

**Figure 1:**
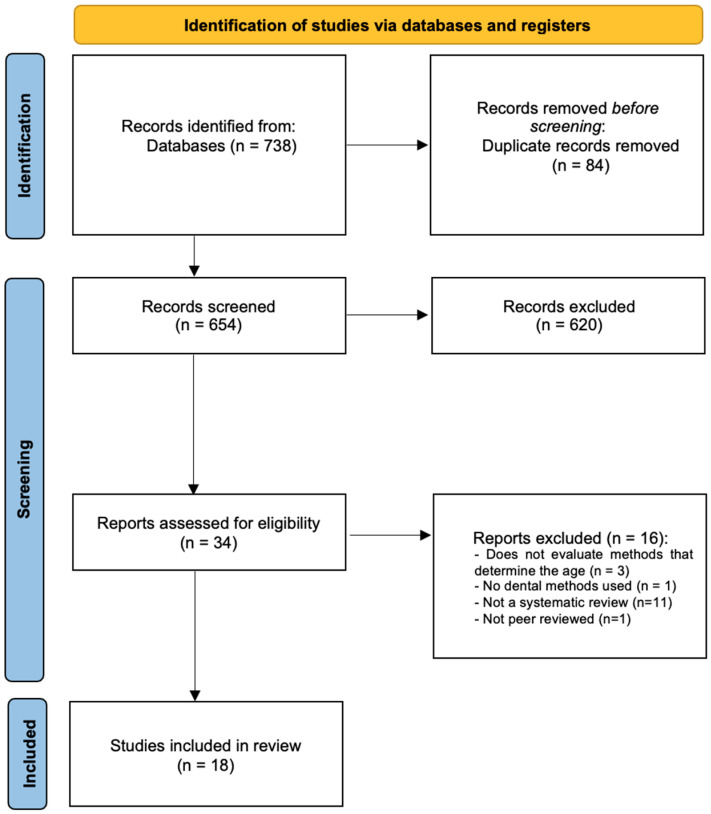
PRISMA flowchart of included studies.

A total of 738 titles were identified by the electronic search. Thirty-four potentially eligible full texts were screened after manual assessment of the title/abstract and deletion of duplicates (Figure 1). During the full-text screening, 16 studies were excluded with justification (Supplementary File S2). A total of 18 systematic reviews met the inclusion criteria. The inter-rater reliability of the full-text screening process was found to be high (kappa score = 1.00).

SR characteristics

Overall, fourteen SRs [6,17,18,19,20,21,22,23,24,25,26,27,28,29] with meta-analysis and four without [30,31,32,33] were included (Table 1). Multiple sub-topics were investigated, such as imagiology methods based on panoramic X-rays [6,19,20,21,22,24,26], Cone Beam Computer Tomography (CBCT), Computer Tomography (CT) [30,32] and Magnetic Resonance Imaging (MRI) [17,30] (Table 1).

One SR failed to report a defined timeframe [33]. Seven SRs described their studies eligibility criteria in the review [20,24,25,30,31,32,33], while four could not describe in adequate detail [18,19,22,30], and eight had no funding information [17,18,23,26,28,29,30,32].

Methodological Quality

We observed excellent inter-examiner reliability at the RoB assessment (kappa score = 0.93; 95% confidence interval: 0.91–0.95).

None of the included SRs fully satisfied the AMSTAR2 Criteria (Table 2). Four studies were rated as of ‘high quality’ [6,21,26,27], three as of ‘moderate quality’ [17,22,25], six as of ‘low quality’ [18,20,28,29,31,33] and five as of ‘critically low quality’ [19,23,24,30,32].

All studies did not report the sources of funding for the studies included in the review, when analyzing the major inconsistencies identified by AMSTAR2 (100%, n = 18) [6,17,18,19,20,21,22,23,24,25,26,27,28,29,30,31,32,33]; 44.4% did not have data selection in duplicate (n = 8) [18,22,23,24,25,28,30,32]; 38.9% of studies did not explain selection literature search strategy (n = 7) [19,24,25,30,31,32,33]; and 33.3% did not have study selection in duplicate (n = 6) [18,22,23,24,30,32].

Synthesis of Results

Overall, three main topics of research were found among the included SRs: panoramic radiographs-based methods; three-dimensional imaging methods; and artificial intelligence (AI)-based methods.

Panoramic radiographs-based methods

Overall, the level of evidence of the SRs focusing forensic methods based on panoramic radiographs was of low quality. Three main methods were the aim of research: Demirjian’s [6,20,21,22,23,24,26,33], Willems’ [21,22,25,26,28,29] and Cameriere’s [19,22,27,32].

As regards to Demirjian’s method, all studies are in agreement of an overestimation that varies between 4 to 9 months [6,20,21,22,24,26]. The majority affirm that this method is geographically sensitive [6,20,21,24], except the studies that are single-population oriented [22,26]. With respect to sex, two studies tend to overestimate the females [21,24], two showed more overestimation in males and lastly, one study reported that there were no differences between sexes [6] and one did not analyze sex subgroups [22]. Demirjian’s method was also applied solely to the third molar in two papers. Haglund et al. [23] defined the accuracy of the method for the 18 years old threshold as 71% and Rolseth et al. [33] identified that the different development ranged from 4 to 7 years of the 3rd molar mineralization.

As for the Willems’ method, most studies showed a slight age overestimation, varying between 1 to 5 months [21,22,25,28,29], aside from one [26], that concluded underestimation by a month. They also conclude that males are more susceptible to this overestimation [25,26,28,29], except one [21], which concludes that females are more sensitive. One other study [22] does not analyze sex subgroups. Respecting age and geographic subgroups, some authors [21,25,28,29] reported differences between populations as the rest only studied a single population [22,26]; Esan et al., Yosuf et al. and Wang et al., also stated that a few age subgroups are more prone to overestimation [21,25,29].

Regarding Cameriere’s method, studies demonstrated overestimation that varied from 3 months to one year, making this variation slightly greater in males, but without statistically significant difference [19,22,32]. Marroquin et al. [32] also reported that in the Indian subpopulation, this variation can be as high as 10 years of overestimation. Cameriere also developed an index to assess the threshold of 18 years old with a percentage of correct classification ranging from 72 to 96%, with a better accuracy in males [27].

Kvall’s method [32] overestimates age between 1 to 2 and 12 to 13 years old and Chaillet’s method [18] within 6 to 8 months. Both methods overestimate more females and present different results when analyzing various geographical subpopulations.

Other methods included in this overview were only investigated by Franco et al. [22] for the Brazilian population. Nolla’s method demonstrated 2 to 3 months of overestimation, Lilequist and Lundberg’s method 1 to 2 months of underestimation, Mornstard’s 3 to 4 months of overestimation and lastly, Haavikko’s method underestimates between 10 to 12 months.

Three-dimensional imaging methods

Overall, the level of evidence of the SRs relating to forensic methods based on 3-dimensional imaging was of low quality.

Cone Beam Computed Tomography (CBCT) and Computed Tomography (CT) Scans reported different margin of errors, according to the method applied, ranging from 3,5 to 28 years [32]. When using pulp/tooth ratio, individuals were correctly identified between 30 and 90%, being the majority around 60% [30]. Both studies [30,32] agree that these values differ depending on the sex of the individual and the type of the tooth.

As for Magnetic Resonance Imaging (MRI), all studies concluded that results from images obtained through MRI are equated to panoramic radiographs, without the ionizing radiation [17,30].

AI-based methods

The level of evidence on AI-based methods for sex prediction using dental measures was collectively based on low quality SRs. AI displayed precision and accuracy similar to trained examiners, overcoming the observer subjectivity. Although the accuracy, real-life testing and validation are yet to be proved [31].

## 4. Discussion

This umbrella review was able to sum up the evidence provided by the available SRs on age estimation methods in Forensic Dentistry. The collective knowledge is currently based on low- to moderate-confidence evidence-based studies, at best ranging from critically low to high quality. Overall, these results show that, due to the poor quality of many of the studies mentioned here, some of the forensic tools used today may be outdated or misused, and some results must be analyzed with caution.

Age estimation is a major step in forensic and archeological investigations [4]. Despite several methods that have been developed to this end, approaches using dental tissues are among the most useful and reliable due to their low changeability and greater resistance to degradation [7,13]. Most studies rely on methods developed upon bidimensional radiographic images, such as Demirjian’s and Willems’ methods, which have seen validation worldwide [6,18,19,20,21,22,24,25,26,27,28,29,32,33]. Both methods tend to overestimate age [6,20,21,22,23,24,25,26,28,29,33]. Cameriere’s method was first studied for several age groups and later as an index (I3M) that differentiates the legal threshold of 18 years old [19,22,27,32]. Other methods included are Chaillet’s, Lundberg’s, Nolla’s, Mornstad’s, Haavik’s and Kval’s methods [18,22,32]. More recently, a few studies investigated the applicability of 3-dimensional imaging in forensic dentistry [17,30,32]. AI technology has already been tested for age estimation and seems like a potential forensic tool [31].

Demirjian’s method obtained global acceptance and became the most widely used method for dental age estimation [21]. Nonetheless, most studies concluded that this method is geographically sensitive and varies according to the subpopulation studied [6,20,21,24]. A possible reason for such a thing was the origin of this dataset (Caucasian subpopulation) with low heterogeneity, different from the other subpopulations studied [6,20,21,24]. Demirjian’s method tends to overestimate the age of the individuals, regardless of the sex of the subject. For such reasons, Demirjian’s method renders as a poor forensic tool when misapplied [20,24]

Willems’ method is also geographically sensitive [21,25,28,29]. Sehrawat et al. [28], for instance, determined that this method overestimates in the majority of the countries, except China and India. Sex seems to be a factor to consider, because this method tends to overestimate more males than females [21,25,26,28,29].

Three studies comparing both Demirjian and Willems’ methods are all in agreement that the latter is more accurate and less prone to overestimation [21,22,26].

Demirjian’s method was introduced in the 1970’s and Willem’s method in the 2000’s. Since these methods (and most of the remaining methods) are based on tooth maturation and this characteristic is growth dependent, it is likely that it will need to be updated on a regular basis. Growth patterns have evolved with the improvement of healthcare, nutrition and genetics so new methods must be developed to accompany the evolution of times [21,34].

Not only in Forensic Dentistry, dental methods and indexes have been misused. In Orthodontics, indexes such as Bolton, developed from a specific subpopulation, have been proven incorrect when generalized to other populations [35].

Cameriere’s method is one of the geographically stable methods and tends to overestimate by 4 months, both boys and girls, without statistically significant difference [19,22,32]. According to Hostiuc et al. this method seems to outperform several others, including Dejirmijian and Willems methods [19].

Santiago et al. concluded that the I3M has been validated in several sub-populations throughout the world, since it has a high accuracy in discriminating whether a person has reached the age of 18 years, regardless of the population studied. In terms of sex, men tend to have better results, but women also have high accuracy, sensitivity and specificity. [27].

Forensic age estimation based on dental measures has been, until recently, based on bidimensional imaging. Three-dimensional imaging is gaining more relevance in all dentistry areas, including forensic dentistry with the first study that reported the use of this technology was published in 2004 [36]. CBCT and CT scans reconstructions allow the investigators to analyze the pulp/tooth volume ratio. The volume ratio might be an interesting tool for predicting age after root maturation of the third molar, around the second decade of life [30]. Despite Micro-CT scans requiring the use of extracted teeth, the images are of greater quality and produce accurate measures because of more spatial resolution that of a CBCT; however, its application in live subjects is not viable, and a model based in this type of imaging may not be replicable for forensic proposes in live individuals [30,32]. Major limitations of this method are the artefacts produced by adjacent metal structures and restorations, such as implants and amalgam fillings [30]. Moreover, the difficulty in reproduction of the site for measurements might lead to an inaccurate analysis; the lack of a simple method of investigation should be the focus for the next researchers [30].

Due to the ethical implications of the usage of ionized radiation for other than diagnostic indications, MRI arose as a valid alternative since it uses strong magnetic fields and radio waves to generate imagens. It is a relatively new tool in age estimation, published for the first time in 2015 [37]. Regarding the methodology itself, MRI can be used associated with other methods previously stated, such as Demirjian’s. MRI tends to be more accurate in the early stages of development but to be more challenging and inaccurate in the latter stages because of the lack of contract between dental and bone tissue. It also takes more time and is more expensive than an ordinary panoramic radiograph [30]. Due to the scarcity of research on this subject, the inter-ethnic variability is not yet proven [17]. Discrepancies between the MRI approaches make it inappropriate to pool data together and perform a proper systematic review with meta-analysis. Furthermore, future age estimation methods based on MRI will probably be based on multifactorial sites and measures [17].

AI-based automated systems have been developed to surpass the examiner’s subjectivity. The AI model that best performed was the Deep Learning Convolutional Neural Network approach, with similar accuracy when compared with trained researchers. AI models that combine a dual Convolutional Neural Network, first to predict sex and then age, outperform a single Convolutional Neural Network approach. However, AI-based models have not proven themselves in the field to be routinely applied [31].

The TRIPOD [38] checklist should be followed for the improvement of research quality. Particular attention should be paid to the choice of study design and reasons for exclusion, and the research question should be more clearly formulated. In order to avoid biases, future SRs should consider the RoB of individual trials and the number of authors who performed data extraction.

Strengths and limitations

There are several strengths of the present umbrella review. Overall, using a transparent and evidence-based methodology, these results provide a comprehensive overview of the available SRs for age determination in Forensic Dentistry. Because the individual studies included in each of the present SRs were not reviewed, we recommend a cautious interpretation. Therefore, the conclusions rely on the interpretation of the authors of the systematic review.

## 5. Conclusions

Current evidence on forensic dentistry methods for age estimation is supported by low- to moderate-confidence systematic reviews. Willems’ method is more accurate and less prone to overestimation. Most methods seem to be geographically sensitive, despite some authors attributing this heterogeneity to methodological errors. Cameriere’s index has high accuracy, regardless of the population studied. Three-dimensional imaging and AI technology, although on the rise, still lack field validation. This umbrella review reports the most common mistakes performed in SRs and will pave the way for more robust evidence-based research in the future.

## Figures and Tables

**Table 1:** Characteristics of included SRs.

Authors (Year)	N	Search Period	Interventions	Quality Assessment Tool	Sample	Method of Analysis	Outcomes	AMSTAR2 Score *	Funding
Bjork (2018) [30]	27	From July 2004 to September 2017	CT and MR imaging	NI	RCTs	SR	Although more research is needed, both CT and MR imaging may be useful tools for age estimation.	Critically Low	NI
De Tobel (2020) [17]	55	Up to September 2018	MR imaging	EPOC overview and QUADAS-2	1 prospective cohort; 35 prospective CS; 19 retrospective CS	SR/MA	The performance of the age estimation was better for the multi-factorial age estimation than for the single-site age estimation. MRI allows the examination of multiple anatomical sites without the use of ionizing radiation.	Moderate	NI
Diaconescu (2021) [18]	25	From 2013 to 2019	Chaillet’s method	STROBE	RCTs	SR/MA	Chaillet’s method showed age overestimation in both sexes, as in most ethnic groups, with delayed dental development in Asian populations, unlike European populations.	Low	NI
Esan (2017) [21]	28	Up to 28 December 2016	Demirjian’s and Willems’ methods	STROBE	5 comparative CS; 4 CS; 17 retrospective CS; 2 observational CS	SR/MA	While Demirjian’s method has wide application in determining maturity scores, Willems’ method provides a more accurate estimate of chronological age in different populations.	High	None
Franco (2020) [22]	13	Up to January 2019	Demirjian, Willems, Cameriere’s, Nolla’s and Lilequist and Lundberg’s methods	JBI Critical Appraisal Tools	CS studies	SR/MA	Optimal performance was achieved by most of the international methods for dental radiographic age estimation.	Moderate	Research Grant
Haglund (2018) [23]	24	Up to 8 June 2017	Demirjian’s method for the 3rd molar	QUADAS-2	RCTs	SR/MA	In addition to the fact that the presence of an immature third tooth is highly indicative of adult age, there are significant numbers of young adults (over the age of 18) with immature third teeth.	Critically Low	NI
Hostiuc (2021) [20]	89	From 1973 to 2020	Demirjian’s method	STROBE	RCTs	SR/MA	Using the Demirjian method, the age is overestimated by about half a year for both sexes. Some geographic/ethnic differences exist. Nevertheless, regardless of the ethnic profile of the subjects, this method is useful.	Low	None
Hostiuc (2021) [19]	15	From 2005 to 2019	Cameriere’s method	STROBE	RCTs	SR/MA	At least in the 7–14 age interval, the Cameriere method is accurate enough for clinical use. However, it should not be used outside of this age range.	Low	None
Jayaraman (2013) [24]	34	From January 1973 to December 2011	Demirjian’s method	NI	RCTs	SR/MA	Demirjian’s method overestimates the age of the subjects by more than six months, and therefore this data set should only be used with great caution when trying to estimate the age of a group of subjects in any global population.	Critically Low	None
Khanagar et al. (2021) [31]	8	From January 2000 to June 2020	AI based models for personal age estimation	QUADAS-2	NR	SR	The AI technology demonstrates an accuracy and precision that is equivalent to that of a trained examiner, overcoming human error and being non-invasive are additional advantages of these models. A major limitation of the present review is the lack of real-life scenarios and the experimental nature of the included studies.	Low	Research Grant
Marroquin (2017) [32]	32	From January 1995 to July 2016	Cameriere’s, Kvaal’s method, and CBCT imaging	NI	NR	SR	More accurate results were obtained with age estimation methods based on pulp/tooth area ratio calculation. It is advised to use dental age estimation methods, firstly, pulp/tooth area ratio calculation of single first, upper canines and other single rooted teeth, and secondly, pulp/tooth length/ratio calculation.	Critically Low	NI
Yusof (2017) [25]	23	From January 2001 to September 2014	Willems’ method	Cochrane handbook for systematic reviews-methodology review	NR	SR/MA	Given the accuracy of the Willems method across different populations, investigators, and age groups, it is appropriate to use this method to estimate age in children.	Moderate	Research Grant
Prasad (2019) [26]	20	Up to July 2018	Demirjian’s and Willems’ methods	QUADAS-2	NR	SR/MA	Willems’ method was more accurate in predicting chronological age than Demirjian’s method in the Indian population, regardless of sex.	High	NI
Rolseth (2018) [33]	21	Up to May 2016	Demirjian’s method for the 3rd molar	QUADAS-2	NR	SR	The differences in the timing of the developmental stages of the third molars according to Demirjian have often been interpreted as differences between populations and ethnic groups.	Low	None
Santiago (2017) [27]	15	Up to November 2017	Cameriere’s method (I3M)	QUADAS-2	CS studies	SR/MA	The Third Molar Maturity Index is a suitable and useful method for estimating adulthood because it has a high accuracy in distinguishing whether an individual has reached 18 years of age, regardless of the population studied.	High	None
Sehrawat (2017) [28]	31	From 2001 to January 2017	Willems’ method	NI	CS and Retrospective studies	SR/MA	Compared to other methods reported in the available literature, the Willems method of dental age estimation results in comparatively less overestimation of age.	Critically Low	NI
Wang (2017) [29]	11	Up to 28 February 2017	Willems’ method	NOS	CS and Retrospective studies	SR/MA	For both sexes, the Willems method overestimated dental age between 3.0 and 16.9 years in almost all age groups. In addition, the accuracy of the Willems method was also shown to be affected by ethnic differences.	Low	NI
Yan (2013) [6]	26	Up to 12 July 2013	Demirjian’s method	STROBE	CS and Retrospective studies	SR/MA	The fact that Demirjian’s method overestimates actual chronological tooth age highlights the need for population-specific standards to better estimate the rate at which human teeth mature.	High	None

AI—artificial intelligence; CBCT—cone beam computer tomography; CS—Cross-sectional; CT-computer tomography, EPOC—effective practice and organization of Care; JBI—Joanna Briggs Institute; MA—meta-analysis; MR- magnetic resonance; N—number of included studies; NI—no information; NOS—Newcastle-Ottawa Scale; NR—not reported; QAS—quality assessment tool. QUADAS—quality assessment and diagnostic accuracy tool; RCT—randomized controlled trials; SR—systematic review; STROBE-Strengthening the reporting of observational studies in epidemiology. * Detailed information regarding the methodological quality assessment is present in Table 2.

**Table 2:** Methodological quality of the included SRs.

First Author	1	2	3	4	5	6	7	8	9	10	11	12	13	14	15	16	Review Quality
Bjørk et al. (2018) [30]	Y	PY	N	N	N	N	N	N	0/0	N	0/0	0	Y	N	0	Y	Critically low
De Tobel et al. (2020) [17]	Y	PY	Y	Y	Y	Y	Y	PY	0/0	N	0/0	0	Y	Y	0	Y	Moderate
Diaconescu et al. (2021) [18]	Y	PY	Y	PY	N	N	PY	N	N/0	N	Y/0	Y	Y	Y	Y	Y	Low
Esan et al. (2017) [21]	Y	Y	Y	PY	Y	Y	Y	PY	PY/PY	N	Y/Y	Y	Y	Y	Y	Y	High
Franco et al. (2020) [22]	Y	Y	Y	PY	N	N	Y	N	PY/PY	N	Y/Y	Y	Y	Y	Y	Y	Moderate
Hadlund et al. (2018) [23]	Y	PY	Y	PY	N	N	Y	PY	N/N	N	Y/Y	Y	Y	Y	Y	Y	Critically low
Hostiuc et al. (2021) [20]	Y	Y	Y	N	Y	Y	Y	PY	N/0	N	Y/0	Y	Y	Y	Y	Y	Critically low
Hostiuc et al. (2021) [19]	Y	PY	N	Y	Y	Y	PY	N	N/0	N	Y/0	Y	Y	Y	Y	Y	Low
Jayaraman et al. (2013) [24]	Y	N	N	N	N	N	Y	PY	N/0	N	N/0	N	N	N	N	Y	Critically low
Khanagar et al. (2020) [31]	Y	PY	N	PY	Y	Y	N	Y	PY/PY	N	0/0	0	N	N	0	Y	Low
Marroquin et al. (2017) [32]	Y	PY	N	PY	N	N	Y	PY	N/N	N	0/0	0	N	N	0	N	Critically low
Mohd Yusof et al. (2017) [25]	Y	Y	N	PY	Y	N	Y	PY	Y/Y	N	Y/Y	Y	Y	Y	Y	Y	Moderate
Prasad et al. (2019) [26]	Y	Y	Y	Y	Y	Y	PY	Y	Y/Y	N	Y/Y	Y	Y	Y	Y	Y	High
Rolseth et al. (2018) [33]	N	Y	N	PY	Y	Y	N	PY	Y/Y	N	0/0	0	Y	Y	0	Y	Low
Santiago et al. (2017) [27]	Y	Y	Y	Y	Y	Y	Y	Y	Y/Y	N	Y/Y	Y	Y	Y	Y	Y	High
Sehrawat et al. (2017) [28]	Y	PY	Y	PY	Y	N	PY	Y	N/N	N	Y/Y	N	N	Y	N	Y	Low
Wang et al. (2017) [29]	Y	Y	Y	PY	Y	Y	PY	Y	Y/Y	N	Y/Y	Y	Y	Y	N	Y	Low
Yan et al. (2013) [6]	Y	Y	Y	Y	Y	Y	Y	Y	Y/Y	N	Y/Y	Y	Y	Y	Y	Y	High

0—No meta-analysis conducted, N—No, Y—Yes, PY—Partial Yes. 1. Are research questions and inclusion criteria included? 2. Were review methods established a priori? 3. Is there an explanation of the review authors’ selection literature search strategy? 4. Did the review authors use a comprehensive literature search strategy? 5. Was study selection performed in duplicate? 6. Was data selection performed in duplicate? 7. Is the list of excluded studies and exclusions justified? 8. Is the description of the included studies in adequate detail? 9. Is there a satisfactory technique for assessing the risk of bias (RoB)? 10. Is there a report on the sources of funding for the studies included in the review? 11. If meta-analysis was performed, did the review authors use appropriate methods for statistical combination of results? 12. If meta-analysis was performed, did the review authors assess the potential impact of RoB? 13. Was RoB accounted for when interpreting/discussing the results of the review? 14. Did the review authors provide a satisfactory explanation for, and discussion of, any heterogeneity observed in the results of the review? 15. If they performed quantitative synthesis, was publication bias performed? 16. Did the review authors report any potential sources of conflict of interest, including funding sources?

## Data Availability

Not applicable.

## Supplementary Materials

The following supporting information can be downloaded at: https://www.mdpi.com/article/10.3390/medicina60010042/s1, Table S1: PRISMA 2020 Checklist; Table S2: Detailed list of excluded articles with reasons.
