# Supplementary material for: Evidence of Age Estimation Procedures in Forensic Dentistry: Results from an Umbrella Review
Source: Medicina (Kaunas). 2023 Dec 25;60(1):42. doi: 10.3390/medicina60010042 (PMC10818637; doi:10.3390/medicina60010042)
Supplement: Supplementary file 1 [file medicina-60-00042-s001.zip › Supplementary file S2.pdf]

Supplementary File 2. Detailed list of excluded articles with reasons.

#	Author	Title	Reference	Reason for exclusion
1	Capitaneanu et al.	A systematic review of odontological sex estimation methods	J Forensic Odontostomatol. 2017 Dec 1;35(2):1-19.	No age estimation
2	Matsuda et al.	Forensic odontology with digital technologies: A systematic review	J Forensic Leg Med . 2020 Aug;74:102004. doi: 10.1016/j.jflm.2020.102004. Epub 2020 Jul 1.	No age estimation
3	Perinetti et al.	The diagnostic performance of dental maturit for identification of the circumpubertal growth phases: a meta-analysis	Prog Orthod. 2013 May 23;14:8. doi: 10.1186/2196-1042-14-8.	No age estimation
4	Dallora et al.	Bone age assessment with various machine learning techniques: A systematic literature review and meta-analysis	PLoS ONE 14(7): e0220242. https://doi.org/10.1371/journal.pone.0220242	No dental methods evaluated
5	Birchler et al.	Dental age assessment on panoramic radiographs in a Swiss population: a validation study of two prediction models	Dentomaxillofac Radiol 2016; 45: 20150137.	Not a systematic review
6	Birchler et al.	Dental age assessment on panoramic radiographs: Comparison between two generations of young Finnish subjects	Journal of International Medical Research 2019, Vol. 47(1) 311–324	Not a systematic review
7	Chudasama et al.	Dental age assessment (DAA): A study of a Caucasian population at the 13 year threshold	Journal of Forensic and Legal Medicine 19 (2012) 22-28	Not a systematic review
8	De Donno et al.	Dental age estimation: Demirjian's versus the other methods in different populations. A literature review	Medicine, Science and the Law 2021, Vol. 61(1S) 125–129	Not a systematic review
9	Jayaraman et al.	Dental age assessment of southern Chinese using the United Kingdom Caucasian reference dataset	Forensic Sci Int. 2012 Mar 10;216(1-3):68-72. doi: 10.1016/j.forsciint.2011.08.019. Epub 2011 Sep 25.	Not a systematic review
10	Jayaraman et al.	Development of a Reference Data Set (RDS) for dental age estimation (DAE) and testing of this with a separate Validation Set (VS) in a southern Chinese population	Journal of Forensic and Legal Medicine 43 (2016) 26-33	Not a systematic review
11	Mitchell el al.	Dental age assessment (DAA): Reference data for British caucasians at the 16 year threshold	Forensic Science International 189 (2009) 19–23	Not a systematic review
12	Peiris et al.	Dental Age Assessment: a comparison of 4- to 24-year-olds in the United Kingdom and an Australian population	International Journal of Paediatric Dentistry 2009; 19: 367–376	Not a systematic review
13	Roberts et al.	Dental age assessment (DAA): a simple method for children and emerging adults	Br Dent J. 2008 Feb 23;204(4):E7; discussion 192-3. doi: 10.1038/bdj.2008.21. Epub 2008 Jan 18.	Not a systematic review
14	Wong et al.	Northern Chinesedentalages estimated fromsouthern Chinese reference datasets closely correlatewith chronological age	Heliyon. 2016 Dec; 2(12): e00216.	Not a systematic review
15	Yadava et al.	Dental age assessment (DAA): reference data for British children at the 10-year-old threshold	Int J Legal Med (2011) 125:651–657	Not a systematic review
16	Rolseth et al.	Demirjian's Development Stages on Wisdom Teeth for Estimation of Chronological Age: A Systematic Review	Oslo, Norway: Knowledge Centre for the Health Services at The Norwegian Institute of Public Health (NIPH); 2017 Mar 15.	Public report not peer reviewed
